# Disseminated coccidioidomycosis mimicking malignant lymphoma in a 14-year-old female

**DOI:** 10.1007/s00292-025-01509-8

**Published:** 2025-11-13

**Authors:** Iris E. Lee, Jun Wang

**Affiliations:** Department of Pathology and Laboratory Medicine, Loma Linda University Health, 92354 Loma Linda, CA USA

**Keywords:** *Coccidioides*, Cocci, Fungi, Mimicker, Endemic, *Coccidioides*, Cocci, Fungi, Imitator, Endemisch

## Abstract

Coccidioidomycosis is a fungal infection that is often asymptomatic in immunocompetent individuals. When symptoms do occur, they often resemble a mild, flu-like illness. However, this disease can become clinically severe and disseminate, especially in immunocompromised patients or other high-risk groups. Failure to consider coccidioidomycosis in the differential diagnosis may lead to missed or delayed diagnosis, resulting in postponement of appropriate treatment.

## Abstract (de)

Die Kokzidioidomykose stellt eine Pilzinfektion dar, die bei immunkompetenten Personen häufig asymptomatisch verläuft. Wenn doch Symptome auftreten, ähneln sie oftmals einem leichten grippalen Infekt. Diese Erkrankung kann jedoch klinisch schwer und disseminiert verlaufen, insbesondere bei immunkompromittierten Patienten oder anderen Hochrisikogruppen. Wird die Berücksichtigung der Kokzidioidomykose in der Differenzialdiagnose versäumt, kann dies zu einer Fehldiagnose oder verspäteten Diagnosestellung führen, was in einer Verzögerung der adäquaten Therapie resultiert.

The ***Coccidioides*** species are thermally dimorphic fungi that can cause infections ranging from an asymptomatic presentation to disseminated clinical findings. In its disseminated form, coccidioidomycosis can present with clinical features that closely resemble other conditions. Herein, the authors highlight how disseminated coccidioidomycosis can mimic lymphoma, thereby emphasizing the importance of including coccidioidomycosis in the differential diagnosis.

## Introduction

Coccidioidomycosis, also known as “valley fever,” is a fungal disease caused by *Coccidioides immitis* and *Coccidioides posadasii*, two species of fungi that are endemic to arid and semiarid regions of the southwestern United States of America (USA) and Latin America [2, 4, 5]. The infection is usually self-limiting; however, a proportion of cases require antifungal medication and treatment [5, 8]. Most patients who present clinically with this infection have respiratory symptoms [1, 8]. A proportion of individuals may progress to life-threatening severe pulmonary or disseminated disease. If there is lack of clinical suspicion for coccidioidomycosis, it may take longer to arrive at the true diagnosis, and essential treatment may be delayed. Presented below is a case of disseminated coccidioidomycosis that mimicked malignant lymphoma.

**Fig. 1:**
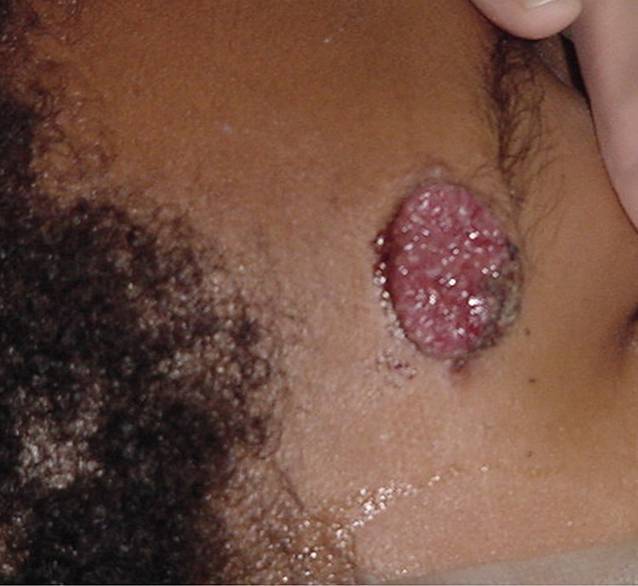
Ulcerated forehead lesion of the 14-year-old female patient

**Fig. 2:**
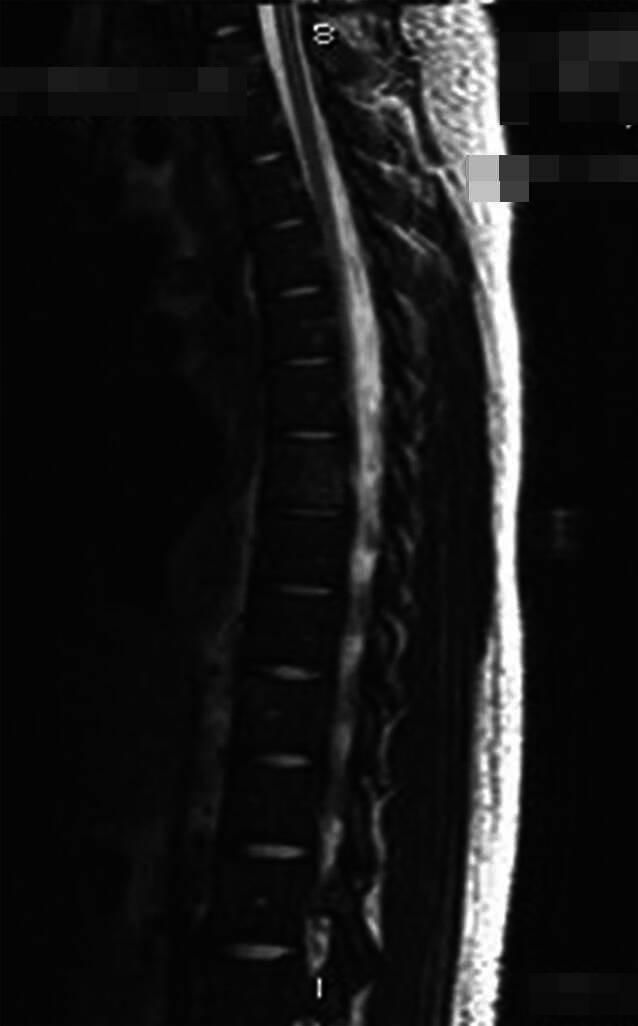
MRI revealing multiple infiltrative lesions involving thoracic and lumbar vertebral bodies

A 14-year-old African American female presented with a 3-month history of shortness of breath, fever, night sweats, and 13.6 kg weight loss. Physical examination showed a superficial forehead skin lesion, and there was no evidence of hepatosplenomegaly (Fig. 1). Computed tomography (CT) and magnetic resonance imaging (MRI) scans revealed diffuse bilateral hilar and mediastinal lymphadenopathy, including multiple infiltrative lesions that involved the thoracic and lumbar vertebral bodies and sacrum (Fig. 2).

**Fig. 3:**
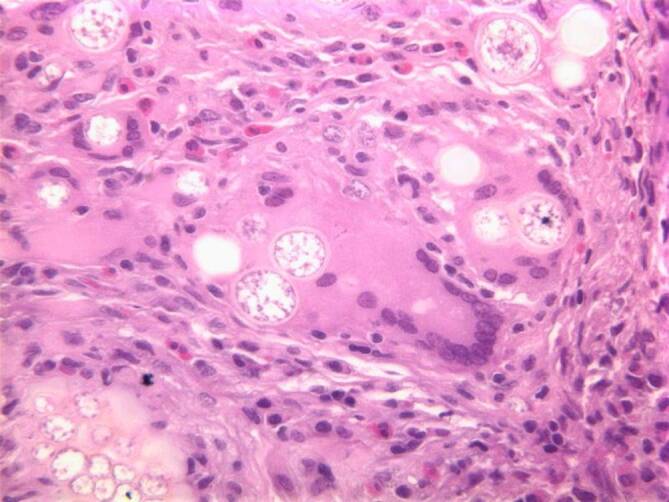
Hematoxylin and eosin (H&E) stain showing a granulomatous giant cell reaction

**Fig. 4:**
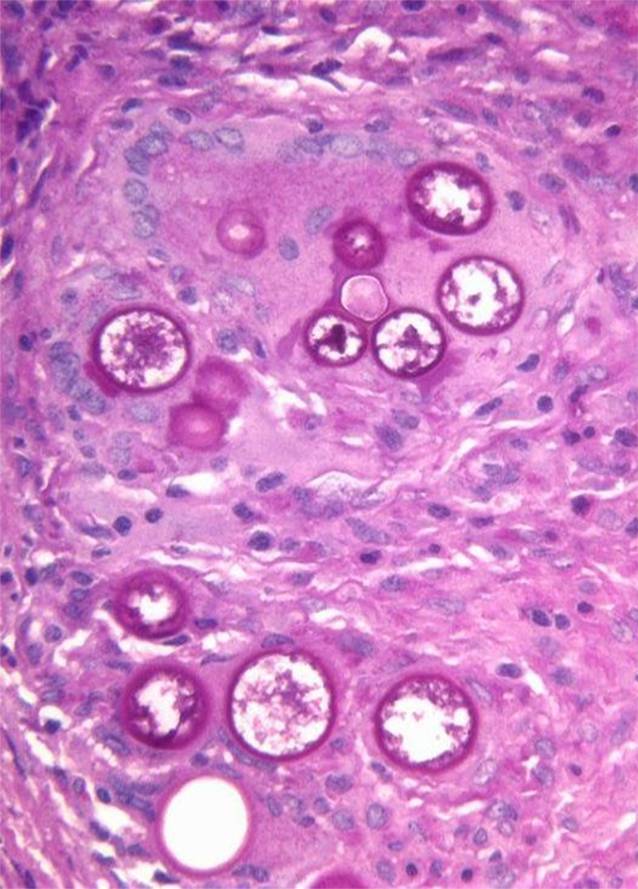
Periodic acid–Schiff stain highlighting thick-walled non-budding spherules filled with small endospores

**Fig. 5:**
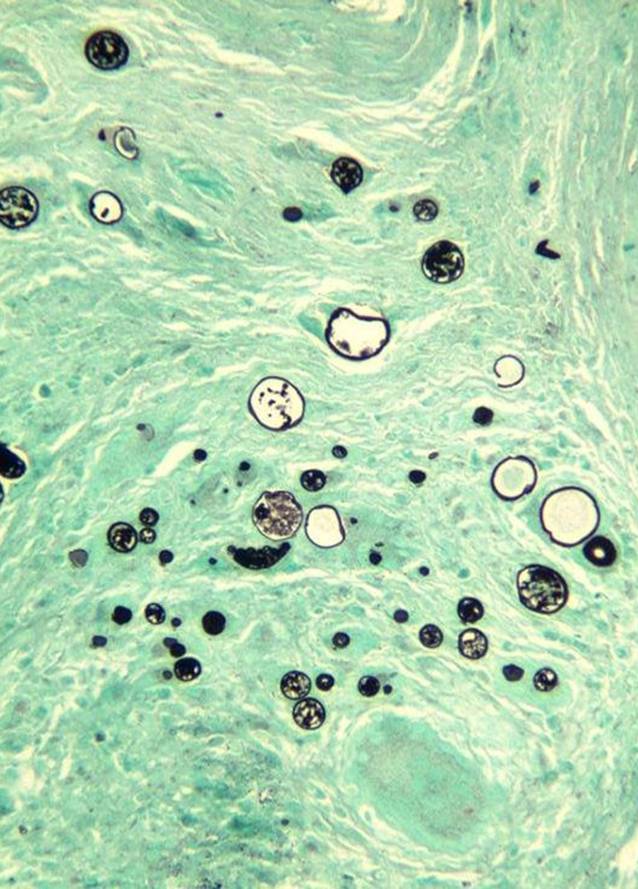
Grocott’s methenamine silver stain highlighting thick-walled non-budding spherules filled with small endospores

**Fig. 6:**
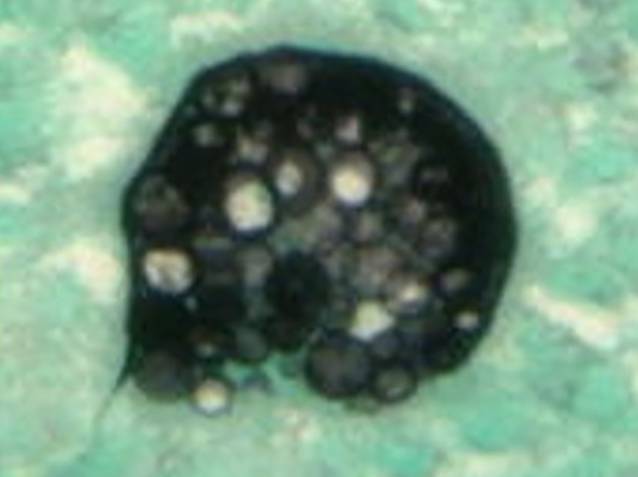
Higher magnification of Grocott’s methenamine silver stain highlighting thick-walled non-budding spherules filled with small endospores

These findings were clinically and radiologically suspicious for lymphoma. The patient underwent bone marrow biopsy to rule out lymphoma, a solid tumor, or an infection with marrow involvement. The bone marrow biopsy was significant for a slightly left-shifted myeloid hyperplasia, mild megakaryocytic hyperplasia, and mild marrow eosinophilia. Significantly, thick-walled non-budding spherules with a granulomatous giant cell reaction were present within the bone marrow trephine core section and stained positively for periodic acid–Schiff (PAS) and Grocott’s methenamine silver (GMS; Figs. 3, 4, 5 and 6). In addition, fungal hyphae with arthrospores were detected in the patient’s blood and bronchial alveolar lavage cultures, and she was conclusively diagnosed with disseminated coccidioidomycosis.

The *Coccidioides* species are soil-dwelling, thermally dimorphic fungi, most common in the southwestern United States, although there appears to be more recent expansion into other central and northwestern states [5, 9]. Recent years have shown an increased disease burden with expansion of the geographic distribution, even though coccidioidomycosis is underdiagnosed and underreported [4, 5, 9]. It is estimated that the actual burden is 10–18 times higher than reported, and these findings are a growing public health concern [4].

*C. immitis* and *C. posadasii* exist as a mycelium in the soil (consisting of filamentous hyphae) and mature into arthroconidia (spores) [6]. The most common method of transmission to humans is by air inhalation of the arthroconidia [2, 7]. In the human body and tissues, the arthrospores transform into spherules, which are filled with hundreds of small endospores; when these spherules rupture, the endospores can disseminate and form new spherules [3, 6].

Approximately 60% of those infected with *Coccidioides* are asymptomatic, with subclinical findings [1, 3, 7]. The other 40% present with flu-like symptoms that are often indistinguishable from pneumonia [3, 8, 9]. This population of patients can present with a broad spectrum of symptoms, including low-grade fevers, headaches, chills, cough, night sweats, weight loss, and/or joint pain [6, 7].

Certain groups of people are at a higher risk of infection and severe disease, especially those who are immunocompromised, such as human immunodeficiency virus (HIV)/acquired immune deficiency syndrome (AIDS) or transplant patients [6]. Other established risk factors include pregnancy and occupations with high exposure to dust and soil [5, 6]. Higher rates of disseminated coccidioidomycosis have been noted in certain racial groups, including African Americans and Filipinos [3, 5, 6]. Persons with disseminated disease most frequently exhibit effects in skin or the musculoskeletal and/or central nervous system [6–8].

There are multiple methods for detecting and diagnosing coccidioidomycosis in affected patients. Detection by culture remains the gold standard for definitive diagnosis [6]. Inoculation of solid culture media will show growth of mycelium/hyphae [2, 6]. In tissue specimens, spherules containing endospores can present microscopically and are highlighted by the special fungal stains PAS and GMS [1, 2, 6, 8]. Serologic antibody testing is frequently used to detect coccidioidomycosis. Immunoglobulin M/G (IgM/IgG) precipitins appear within 1 to 4 weeks of symptom onset [6, 8]. Complement-fixing antibodies occur at lower titer initially but rise if dissemination occurs [6].

Depending on the severity of infection and the clinical history of the patient, treatment may vary. Immunocompetent hosts who are asymptomatic or have mild primary infections may not need any antifungal treatment and can overcome the infection naturally [3, 5]. Triazoles are usually the first line of treatment for symptomatic coccidioidomycosis, with fluconazole favored over other azoles [3, 5, 9]. Amphotericin B is used for persistent lung lesions, disseminated disease, meningitis, and/or cases that are resistant to triazoles [3, 5, 9].

In summary, the clinical presentation and radiological findings of this 14-year-old patient were strongly suggestive of a malignant lymphoma or solid tumor with bone marrow involvement. However, further diagnostic evaluation ultimately led to the diagnosis of coccidioidomycosis. This case underscores the importance of including coccidioidomycosis in the differential diagnosis when evaluating patients with similar presentations, particularly in endemic regions or high-risk populations.

## Practical conclusion

- Coccidioidomycosis is a fungal disease caused by the thermally dimorphic fungi *Coccidioides* species.
- Disseminated coccidioidomycosis is occasionally seen and can present with clinical features suggestive of lymphoma.
- Consider coccidioidomycosis as a differential diagnosis in patients with high-risk features to prevent a delay in appropriate treatment.

## Declarations

For this article no studies with human participants or animals were performed by any of the authors. All studies mentioned were in accordance with the ethical standards indicated in each case.

The supplement containing this article is not sponsored by industry.
